# Adaptation to potassium starvation of wild-type and K^+^-transport mutant (*trk1,2*) of *Saccharomyces cerevisiae*: 2-dimensional gel electrophoresis-based proteomic approach

**DOI:** 10.1002/mbo3.23

**Published:** 2012-06

**Authors:** Samuel Gelis, Miguel Curto, Luis Valledor, Asier González, Joaquín Ariño, Jesús Jorrín, José Ramos

**Affiliations:** 1 Department of Microbiology, University of CórdobaCórdoba, Spain; 2 Department of Biochemistry and Molecular Biology, Agricultural and Plant Biochemistry and Proteomics Research Group, University of CórdobaCórdoba, Spain; 3 Molecular Systems Biology, University of ViennaVienna, Austria; 4 Institut de Biotecnologia i Biomedicina and Departament de Bioquímica i Biologia Molecular, Universitat Autònoma de BarcelonaBellaterra 08193, Barcelona, Spain

**Keywords:** 2D-gels, potassium homeostasis, *Saccharomyces cerevisiae*, *TRK1*, *TRK2*

## Abstract

*Saccharomyces cerevisiae* wild-type (BY4741) and the corresponding mutant lacking the plasma membrane main potassium uptake systems (*trk1,trk2*) were used to analyze the consequences of K^+^ starvation following a proteomic approach. In order to trigger high-affinity mode of potassium transport, cells were transferred to potassium-free medium. Protein profile was followed by two-dimensional (2-D) gels in samples taken at 0, 30, 60, 120, 180, and 300 min during starvation. We observed a general decrease of protein content during starvation that was especially drastic in the mutant strain as it was the case of an important number of proteins involved in glycolysis. On the contrary, we identified proteins related to stress response and alternative energetic metabolism that remained clearly present. Neural network-based analysis indicated that wild type was able to adapt much faster than the mutant to the stress process. We conclude that complete potassium starvation is a stressful process for yeast cells, especially for potassium transport mutants, and we propose that less stressing conditions should be used in order to study potassium homeostasis in yeast.

## Introduction

Alkali metal cations, especially potassium and sodium play an important role in cell physiology and metabolism. Among organisms studied, the yeast *Saccharomyces cerevisiae* is still chosen as a model to elucidate homeostasis in eukaryotic cells because of the availability of the complete genome sequence (Goffeau et al. 1996), an in silico prediction of all transporters (Nelissen et al. 1997), a wide range of genetic tools to generate mutants, and availability of postgenomic tools such as proteomic databases.

In yeasts cells, intracellular concentrations of Na^+^ and K^+^ are around 10–20 and 200–300 mM, respectively. K^+^ is required for many physiological functions, such as cell volume and intracellular pH regulation, maintenance of stable potential across the plasma membrane, protein synthesis, and enzyme activation (Hoffman 1964; Ariño et al. 2010). *Saccharomyces cerevisiae* cells are able to grow in media containing a large range of K^+^, from 2 μM to 2 M, in all conditions internal K^+^ remains quite constant that allows normal cell growth and division (Ramos et al. 1994; Haro and Rodríguez-Navarro 2002).

Two different systems of K^+^ uptake have been described in *S. cerevisiae* (Ramos and Rodriguez-Navarro 1986). A low-affinity mode of transport with a Km in the millimolar range, observed in cells cultured without K^+^ limitation, and a high-affinity transport with a Km in the micromolar range observed in either K^+^-starved cells or cells growing in the presence of Na^+^. Full activity of the high-affinity K^+^ transport is usually observed after growing the cells without K^+^ limitation in minimal medium and then starving the cells during 4–5 h in the same medium lacking added K^+^ (i.e., arginine phosphate medium; Rodriguez-Navarro and Ramos 1984). Active K^+^ uptake is mediated by two membrane transporters, Trk1 and Trk2, Trk1 being the most important (Ko et al. 1990; Ramos et al. 1994). Deletion of both genes leads to a growth inhibition at low K^+^ concentrations, hyperpolarization of plasma membrane, and observation of residual ectopic potassium transport (Madrid et al. 1998; Navarrete et al. 2010). Those phenotypes appear to be due mainly to *TRK1* deletion as the effect of *TRK2* absence is almost negligible in most experimental conditions (Madrid et al. 1998).

Two-dimensional (2-D) gel-based comparative proteomics analyses have been widely used to characterize yeast strains (Usaite et al. 2008; Karhumaa et al. 2009), growth phase (Bruckmann et al. 2009; Cheng et al. 2009; Massoni et al. 2009), or stress responses (Braconi et al. 2009). In our laboratory, we previously focused our proteomic analysis on the double mutant (DM) *trk1,trk2* mutant growing without potassium limitation in exponential and stationary phase (Curto et al. 2010). It was observed that there were almost no differences between wild-type and DM strains at the exponential phase of growth. However, significant differences related mainly to glycolytic enzymes were found at stationary phase.

In this study, a similar kind of analysis was used to characterize the same wild-type and DM *trk1,trk2* in the extreme condition of potassium starvation. Statistically significant differences were observed in the protein 2-D profile, corresponding both to the mutations and/or potassium starvation. Spot intensity values were subjected to uni- and multivariant statistical analyses and a clustering test. Major and variable spots were mass spectrometry (MS) analyzed, and 73 protein species, corresponding to 49 unique gene products were identified. We conclude that potassium starvation is a very stressful condition to study potassium homeostasis, especially in the case of double *trk1,2* mutant strain.

## Results

### 2-D protein profiles

Strains were grown in translucent YNB-F media with no limiting potassium (50 mM KCl) until they reach an OD_600nm_ of around 1.9 in order to obtain high cell biomass but still in exponential phase (Navarrete et al. 2010). Parental strain BY4741 and DM *trk1,trk2* were then transferred to medium without added potassium, samples of cells were taken during 5 h and proteins were extracted. Protein yield obtained after extraction plus TCA–acetone precipitation was evaluated. Cells of both strains kept full viable as monitored by colony forming units counts (9.3 × 10^6^ ± 0.2 and 9.5 × 10^6^ ± 0.3 at time zero in wild-type and DM strains, respectively, and 2.1 × 10^7^ ± 0.3 and 1.1 × 10^7^ ± 0.4 after 5-h starvation), but total protein yield decreased in function of starvation time from 37.55 to 34.20 μg eq. serum albumin bovine mg^−1^ dry weight for the parental strain and from 31.08 to 5.28 μg eq. SAB mg^−1^ dry weight for the mutant strain were obtained (Table 1).

**Table 1:** Optical density, protein yield, and number of spots resolved in 2-DE, with indication of qualitative and quantitative differences of spots regarding the wild-type strain at time 0

						Variable spots (with respect to WT 0)			
						
						Qualitative		Quantitative 1.5-fold	
							
Yeast strain	Time (min)	OD_600nm_ mean SD	DW* (mg)	Protein yield μg eq. SAB/mg DW	Total of consistent spots	Appear	Disappear	Up	Down
BY4741 (WT)	0	1.89 ± 0.04	96.54	37.55	271				
	30	2.02 ± 0.068	103.17	37.34	264	0	15	6	10
	60	2.17 ± 0.076	110.52	37.15	253	0	25	5	15
	180	2.25 ± 0.05	114.75	36.17	248	0	32	2	32
	300	2.28 ± 0.093	116.13	34.20	239	0	33	1	48
BYT12 (*trk1,2*)	0	1.86 ± 0.032	95.01	31.08	208	0	69	11	47
	30	1.80 ± 0.05	91.95	29.23	193	0	95	13	31
	60	1.81 ± 0.051	92.16	16.84	170	0	110	11	21
	180	1.73 ± 0.042	88.38	8.44	138	0	142	9	16
	300	1.67 ± 0.059	85.02	5.28	106	0	162	6	20

* DW = [0.51 × OD_600nm_] mg·mL^−1^ medium.

**Figure 1:**
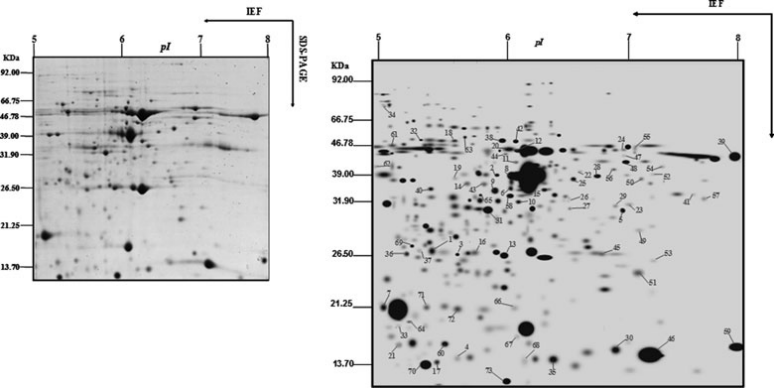
Representative 2-D gel corresponding to extracts from wild cells in control conditions (A), and master gel, with arrows pointing at variable spots (B). Molecular mass (on the left) and p*I* (on the top) were calculated using standard molecular weight markers and the PD-Quest software.

After 2-D electrophoresis and Coomassie staining of the gels, 106–271 consistent spots (present in the three replicates) were resolved in the 5–8 pH range and 10–90 kDa molecular weight (Mr) range (Fig. 1). A 2-D gel image analysis was performed using PDQuest software v 8.01 and, at all times studied, quantitative and qualitative differences in spot intensity were observed between parental and mutant strains. During potassium starvation and comparing to the parental strain at time 0, we observed an increased number of missing spots; thus, 33 spots were missed after 5-h starvation in the case of the parental strain and 162 spots in the case of the *trk1,2* DM (Table 1). Interestingly, new additional spots were not found either in the wild type or in the mutant during the starvation process. Quantitatively, the same behavior was observed in both strains, most spots intensity tends to decrease and just few of them were increased during starvation.

**Figure 2:**
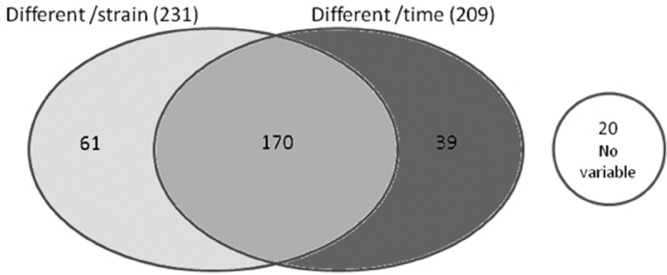
Graphical representation of spots statistically different during potassium starvation in function of strains and/or time after two-way ANOVA and also the 20 spots always present in both strains at any sampled time.

After applying a two-way analysis of variance (ANOVA), 231 spots were assumed to be differentially accumulated between strains and 209 spots between the different sampling times (Fig. 2). A total of 61 spots were variable between strains, but not between sampling times, reflecting those proteins not affected by the experimental environment but by the mutation. On the other hand, 39 spots showed differences only between the different sampling times, reflecting those common responses to the treatment in wild-type and mutant strains. Most of the gel spots (170) presented significant differences between strains and time, showing that the experimental environment affects both wild-type and mutant strains, but in a different way. Moreover, 20 spots were invariable across strains and sampling times (Table S1).

**Figure 3:**
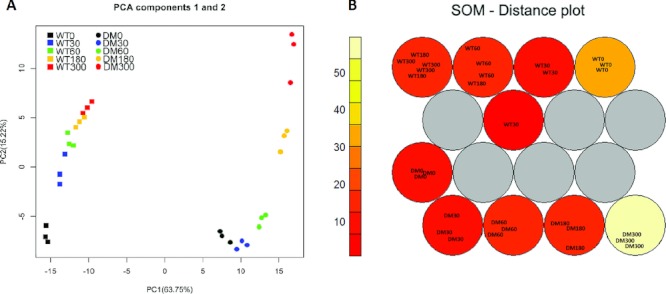
(A) Representation of the samples based on main principal components found after PCA by 2-D plotting of the main principal components (PC1 and PC2). (B) SOM analysis representation. The samples are grouped in nodes based on the scores obtained after applying Kohonen's self-organizing map algorithms. The topology of the grid was set to hexagonal and 4 × 4 and the distances are indicated in left-side bar.

To obtain further information, different and additional statistical approaches were performed. First of all, a data reduction to the whole dataset by means of principal component analysis (PCA) analysis was applied. Of the potential 290 principal components (PC) extracted, the first six PCs accounted for 94.18% of the biological variability (Table 2). The use of these components in a 2-D representation (plotting PC1 and PC2) allowed the effective separation of samples into the different strains and sampling times (Fig. 3). In the DM, duration from 0 to 60 min were closely grouped in both plots, indicating similarity in the spot map. The correlation of each particular spot to PC 1 and 2 was determined from the loading matrix generated during the PCA (Tables S2 and S3). The five spots showing the highest correlation with each PC were determined. Of these spots, five (7, 9, 21, 62, and 68) were identified after MS analysis, corresponding to a co-chaperone protein, dihydrooronate dehydrogenase, glutaredoxin-1, S-adenosylmethyonine synthase, and ubiquitin-conjugating enzyme, respectively (see Discussion).

**Table 2:** Proportion of the explained variance and standard deviation of the principal components obtained after performing a principal component analysis, employing whole dataset and the normalized spot intensities of each spot

	PC1	PC2	PC3	PC4	PC5	PC6	PC7	PC8	PC9
Standard deviation	131.319	64.175	409.411	35.391	27.224	211.077	157.055	147.692	136.269
Proportion of variance	0.6375	0.1522	0.06196	0.0463	0.0274	0.01647	0.00912	0.00806	0.00686
Cumulative proportion	0.6375	0.7897	0.85172	0.898	0.9254	0.94189	0.95101	0.95907	0.96594

Neural network-based analysis was performed employing Kohonen's Self Organizing Maps (SOM), known to be a powerful multivariate analysis method, with a mathematical basis completely different than PCA. Wild-type and DM strains were completely differentiated (Fig. 3). In the case of the wild type, samples coming from time 180 min (WT180) and 300 min (WT300) were in the same node, being this node closer to WT60 while WT30 and WT0 were more distant. In the case of DM, nodes grouping samples DM0, DM30, and DM60 were closer together while DM180 and DM300 were clearly more distant.

### MS analysis and protein identification

Seventy-three variable spots from the most abundant were excised and analyzed by MALDI-TOF-TOF MS after trypsin digestion. Results obtained were compared to the UniProt database allowing the identification of 49 unique proteins. Resulting proteins were classified in functional categories and are presented in Table 3; complementary information including indications on location, number of molecules per cell, and the identified peptide sequence is available in Table S4. A good correlation between theoretical and experimental pI was obtained whereas some differences in Mr were observed. For some spots, a higher Mr was observed maybe due to the absence of mature form in UniProt database. We identified proteins with double experimental Mr, including spots 63 (superoxide dismutase, reported as homodimer), spot 16 (FK506 binding protein 1), and spot 38 (hydroxyacylglutathione hydrolase). On the other hand, there were spots with a much lower Mr experimental than theoretical, probably due to degraded proteins. This was the case for the spot 30 (glyceraldehyde-3-phosphate dehydrogenase 3), spot 70 (protein YLR301W), spot 73 (uracil phosphoribosyltransferase), spot 39 (inhibitory regulator protein Bud2), and spot 59 (Sok2). Finally, there was a group of spots corresponding to the same proteins but with different Mr, possibly due to the presence of isoforms. We identified in this group the glyceraldehyde-3-phosphate dehydrogenase 2 (spots 22–24), the glyceraldehyde-3-phosphate dehydrogenase 3 (spots 25–30), the hexokinase 2 (spots 33 and 34), the phosphoglycerate kinase (spots 47–56), and the uracil phosphoribosyltransferase (spots 71 and 72).

**Table 3:** List of identified proteins

Proteins*	Accession number†	Spots‡
Glycolysis		
Enolase 1	P00924/YGR254W/Eno1	10–11–12
Enolase 2	P00925/YHR174W/Eno2	13–14
Fructose-bisphosphate aldolase	P14540/YKL060C/Fba1	18–19
Glyceraldehyde-3-phosphate dehydrogenase 2	P00358/YJR009C/Tdh2	22–23–24
Glyceraldehyde-3-phosphate dehydrogenase 3	P00359/YJL052W/Tdh1	25–26–27–28–29–30
Hexokinase-2	P04807/YGL253W/Hxk2	33–34
Phosphoglycerate kinase	P00560/YCR012W/Pgk1	47–48–49–50–51–52
		53–54–55–56
Phosphoglycerate mutase 1	P00950/YKL152C/Gpm1	57
Triosephosphate isomerase	P00942/YDR050C/Tpi1	65–66
Ethanol fermentation		
Alcohol dehydrogenase 1	P00330/YOL086C/Adh1	6
Pentose phosphate pathway		
6-phosphogluconolactonase 3	P38858/YHR163W/Sol3	58
Methylglyoxal pathway		
Hydroxyacylglutathione hydrolase	Q05584/YDR272W/Glo2	38
Cell wall biogenesis		
Mannose-1-phosphate guanyltransferase	P41940/YDL055C/Psa1	42
Mannose-6-phosphate isomerase	P29952/YER003C/Pmi40	43
Oxidoreductases/redox balancing		
Fumarate reductase	P32614/YEL047C/Frd1	20
NADPH dehydrogenase 2	Q03558/YHR179W/Oye2	44
Phosphate		
Inorganic pyrophosphatase	P00817/YBR011C/Ipp1	40
Glycerol		
(DL)-glycerol-3-phosphatase 1	P41277/YIL053W/Rhr2	1
Pyrimidine		
Dihydroorotate dehydrogenase	P28272/YKL216W/Ura1	9
Uracil phosphoribosyltransferase	P18562/YHR128W/Fur1	72–73
Amino acid biosynthesis		
3′(2′),5′-bisphosphate nucleotidase	P32179/YOL064C/Met22	2
Homocysteine S-methyltransferase 2	Q08985/YPL273W/Sam4	36
Ketol-acid reductoisomerase	P06168/YLR355C/Ilv5	41
Saccharopine dehydrogenase	P38999/YNR050C/Lys9	61
S-adenosylmethionine synthetase	P10659/YLR180W/Sam1	62
Cofactor production		
3,4-dihydroxy-2-butanone 4-phosphate synthase	Q99258/YDR487C/Rib3	3
3-hydroxyanthranilate 3,4-dioxygenase	P47096/YJR025C/Had1	4
Hit family protein 1	Q04344/YDL125C/Hnt1	35
Protein fate		
ADP-ribosylation factor 1	P11076/YDL192W/Arf1	5
Co-chaperone protein Sba1	P28707/YKL117W/Sba1	7
Family of serine hydrolases 1	P38777/YHR049W/Fsh1	15
FK506-binding protein 1	P20081/YNL135C/Fpr1	16
Peptidyl-prolyl cis-trans isomerase	P32472/YDR519W/Fpr2	17
G-protein beta subunit	P38011/YMR116C/Asc1	31
Peptidyl-prolyl cis-trans isomerase	P14832/YDR155C/Cpr1	46
Signaling		
Inhibitory regulator protein Bud2/Cla2	P33314/YKL092C/Bud2	39
Protein Sok2	P53438/YMR016C/Sok2	59
Stress related		
Cytochrome c peroxidase	P00431/YKR066C/Ccp1	8
Glutaredoxin-1	P25373/YCL035C/Grx1	21
Heat shock protein Ssb2	P40150/YNL209W/Ssb2	32
Heat shock protein 26	P15992/YBR072W/Hsp26	37
Peptide methionine sulfoxide reductase	P40029/YER042W/Mxr1	45
Superoxide dismutase	P00445/YJR104C/Sod1	63
Thioredoxin-2	P22803/YGR209C/Trx2	64
Ubiquitin-conjugating enzyme Ubc2	P15731/YBR082C/Ubc2	67
Ubiquitin-conjugating enzyme variant	P53152/YGL087C/Mms2	68
No well-defined functional category/unknown		
Protoplast secreted protein 2	Q12335/YDR032C/Pst2	60
Uncharacterized phosphatase	P53981/YNL010W	69
Uncharacterized protein YLR301W	Q05905/YLR301W/Hri1	70–71

* Functionally related proteins.

† Swiss-Prot and Saccharomyces Genome Database (SGD) accession numbers and common name.

‡ Numbers corresponded to Figure 1.

Within the proteins identified, we found proteins related to metabolism (28 proteins), mainly involved in the glycolytic pathway (nine proteins), to stress response (nine proteins), protein fate (seven proteins), signaling (two proteins), or other functions (three proteins). The different pathways related to energy production were not altered equally during potassium starvation in the two yeast strains. The level of the nine proteins of the glycolysis tend to decrease radically in the mutant *trk1,2* while their level remained constant in the parental strain, except in the case of two proteins (Hxk2 and Tdh2). On the contrary, proteins of the pentose phosphate pathway (Sol3) and the methylglyoxal pathway (Glo2) remained present during the starvation process although a tendency to smoothly decrease was observed in the methylglyoxal pathway in the case of the mutant strain. Interestingly, some biosynthetic pathways seem to remain active during potassium starvation, like the pyrimidine pathway (Ura1 and Fur1) that keeps a relative good level of proteins, and the amino acid biosynthesis-related proteins (Met22, Sam4, Sam1, and Lys9) that present a higher amount of proteins in the mutant strains during potassium starvation. Proteins related with DNA-repair system are found in both strain (Ubc2 and Mms2). In the case of the mutant, they were present with a two-fold factor increase. Proteins related with stress, especially with oxidative stress, presented the same behavior, Sod1 and Ccp1 were expressed in both strains, being more important the amount in the mutant strain. Other proteins such as Trx2 and Grx2 were only detectable in the mutant strain after 30–60 min of potassium starvation. Finally, other proteins showed a pattern difficult to explain with no homogenous behavior within a same functional category or between spots corresponding to the same single proteins (Hri1).

**Figure 4:**
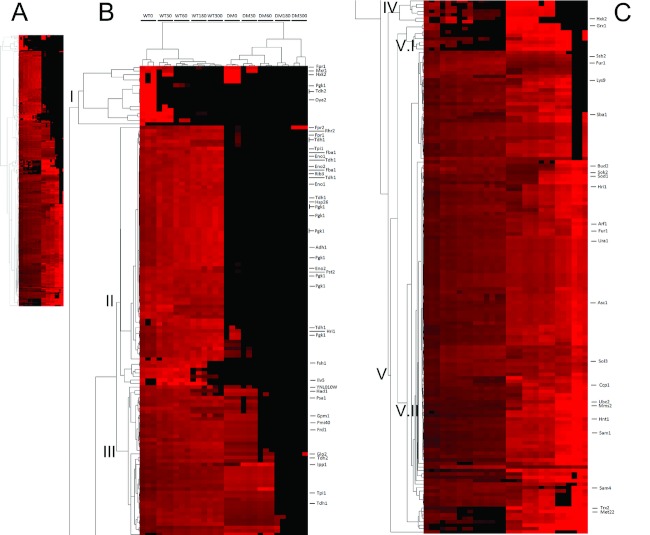
Two-way hierarchical cluster of differentially accumulated spots. A heat map representation of the clustered spots shows the protein value according to the standardized spot percent in each replicate, with red intensity indicating accumulation and black absence. Samples were grouped employing a correlation-based distance. Original plot was divided for a better presentation and reading. (A) Miniature of the original heat map, (B and C) magnified upper and lower parts, respectively, including the names of the identified proteins.

In order to better understand the behavior of the different groups of proteins identified, differential spots were clustered employing Ward's minimum variance method over a Pearson distance-based dissimilarity matrix (Fig. 4), which has proved to be an accurate procedure for proteomics data (Meunier et al. 2007). Spots in the same tree were compared, employing a clustering method and a representation of quantitative variations between strains and times. While wild-type samples showed bigger distance between time 0 and the several starvation times, DM samples were closely grouped from 0 to 60 min suggesting a slower response of the mutant to the potassium starvation process. These results are in agreement with the PCA and SOM analyses shown above.

### Evaluation of hexokinase and alcohol dehydrogenase activity in wild-type and *trk1,2* cells upon potassium starvation

**Figure 5:**
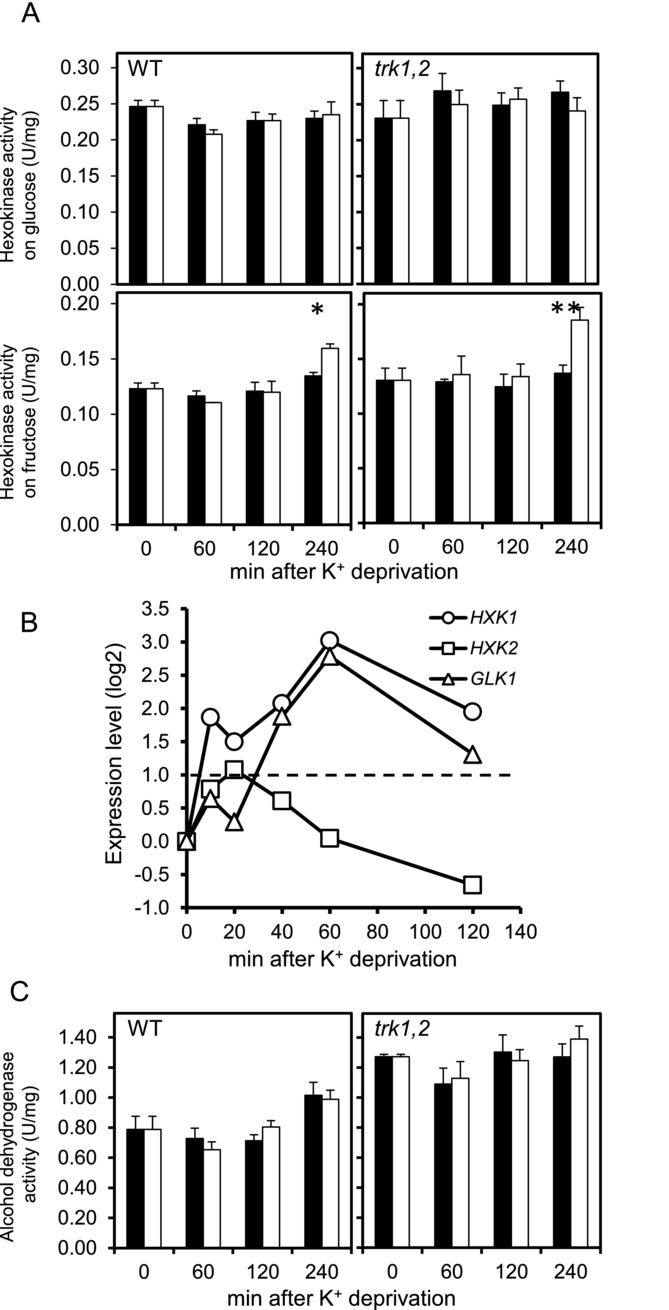
Changes in enzymatic activities triggered by potassium starvation. (A) Determination of hexokinase activity in wild-type and *trk1,2* strains in cells growing in 50 mM K^+^ (closed bars) or in the absence of the cation, (open bars). Substrates are glucose (upper panel) or fructose (lower panel). Data are mean ± SEM from four to eight experiments. *P < 0.05; **P < 0.01. (B) Profile of expression pattern for genes encoding glucose phosphorylation enzymes in response to potassium starvation (no K^+^ vs. 50 mM K^+^). Data were obtained by DNA microarray analysis (Barreto et al., unpubl. ms.) and correspond to the mean from four bio-logical replicates. Values above the discontinuous line are considered significant gene inductions. (C) Determination of alcohol dehydrogenase activity in wild-type and *trk1,2* strains in the presence of absence of external potassium (see A). Data are mean ± SEM from three experiments.

To complement the data obtained by our proteomic analysis, we selected two examples of glycolytic activities corresponding to proteins identified above, the first one is hexokinase, which is an example of activity likely to decrease (due to the loss of Hxk2), and the second is alcohol dehydrogenase (Adh), which is a possible example of unaltered activity. As observed in Figure 5, the hexokinase activity versus glucose or fructose is very similar for wild-type and *trk1,2* cells in the presence of potassium. Interestingly, deprivation of potassium did not decrease the amount of glucose phosphorylation activity, but it resulted in a relative increase in the preference for fructose in wild-type and, even more markedly, in Trk-deficient cells. Although apparently surprising, this result is compatible with the disappearance of Hxk2. There are three glucose-phosphorylating enzymes in yeast: Hxk1, Hxk2, and Glk1. They differ in their *V*_max_ for fructose and glucose, being the fructose/glucose ratio of 3 for Hxk1 and 1.2 for Hxk2 (Barnar 1975). Glk1 barely phosphorylates fructose (Lobo and Maitra 1977). We observe that after 240 min, there is an increase in the fructose/glucose phosphorylation ratio from 0.60 ± 0.03 to 0.73 ± 0.06 in wild-type cells, and 0.52 ± 0.04 to 0.82 ± 0.09 in the *trk1,2* mutant (in which the disappearance of Hxk2 is more prominent; see Table S4). The transcriptomic profile shown in Figure 5 indicates that *HXK1*, encoding the most effective fructose-phosphorylating isoform, is greatly induced by potassium starvation, whereas *HXK2* is not. The emergence of Hxk1 and the disappearance of Hxk2 would explain the increase in the ratio of phosphorylation—fructose/glucose. This increase is probably less drastic than expected because Glk1, whose activity levels are normally lower, is also significantly induced.

In contrast, the amount of alcohol dehydrogenase activity is not decreased by lack of potassium (see Fig. 5), in agreement with the stability of Adh1, the major Adh isoform in exponentially growing yeast (Leskovac et al. 2002).

## Discussion

*Saccharomyces cerevisiae* is endowed with two genes coding for the main plasma membrane potassium transporters. These proteins are essential for the cell to grow at limiting potassium concentrations and mutants lacking the corresponding genes (*TRK1* and *TRK2*) show defective growth and transport at low potassium. However, at high potassium (in the mM range) other no specific systems can transport enough amounts of the cation, thus allowing mutant cells to grow at rates similar to those in the wild type (Navarrete et al. 2010). A first proteomic study of *trk1,2* mutants was recently published (Curto et al. 2010). It is important to notice that in that paper both wild-type and mutant cells were grown under nonlimiting potassium concentrations (50 mM KCl) and the authors concluded that most of the differences observed between parental and DM strains corresponded to proteins related to glycolysis and redox-homeostasis enzymes.

Considering that full activity of the so-called high-affinity/high-velocity potassium transport process dependent upon the Trk1/2 system is usually observed after obtaining K^+^-starved cells by incubation in media without added potassium during 4–5 h (Rodríguez-Navarro and Ramos 1984; Bertl et al. 2003), we decided to study changes in the proteome of the wild type and DM during the starvation process. The most important observation was the extra-ordinary decay in protein content and number of spots, observed during the 5 h of starvation with special relevance in the case of the mutant strain. It has to be considered that in 2D gels, only a fraction of the total proteome can be observed, mainly proteins that are the most abundant such as the housekeeping proteins (Buxbaum 2010). Previous work reported that K^+^ deprivation during 24 h produced, in *S. cerevisiae*, a decrease in cell viability by inducing a programmed cell death process (Lauff and Santa-María 2010). Although this is a very important observation, in our conditions and after 5-h starvation we observed high decrease in protein content but no changes in cell viability. From these results, we conclude that potassium starvation is a very stressing process for the cells and it looks like 5-h incubation without potassium is excessive since it provokes a very important and general decay in many cellular processes. As mentioned above, 4–5 h K^+^-starvation is a general method used to induce full activity of the Trk1/2 system. However, we have recently shown that in the newly designed medium YNB translucent (Navarrete et al. 2010), used also in this work, adaptation to the high-affinity/high-velocity state is much faster. In fact, the higher affinity for Rb^+^ (K^+^) is observed after 30-min starvation and higher *V*_max_ is reached after 2-h starvation. Therefore, we propose that this would be a much more rational way to obtain yeast cells expressing the high-affinity/high-velocity mode of transport.

PCA analysis allowed a clear classification of samples. The plot of PC1 (50.7%) and PC2 (22.2%) shows differences between mutant and wild type and also between the different sampling times. This supports the above-indicated hypothesis in terms of the differences found between strains behavior during the adaptation to the lack of potassium. This idea was confirmed after the application of a SOM neural network, a methodology for the classification of the samples more powerful than PCA analyses. Wild-type strain adapted quicker to the new conditions, since samples taken at 0, 30, and 60 min were already separated. Adaptation seems to be already completed after 3 h since samples taken at 180 and 300 min group together. On the other hand, the DM needed more time to try to adapt to the new environmental condition, that is, samples at time 0, 30, and 60 min grouped together. In conclusion, the wild-type strain adapted and got stabilized faster to the stress condition while the mutant seems to have problems to sense or adapt to the absence of potassium.

It is relevant that in the 2D gels we have identified most of the enzymes involved in glycolysis. During the starvation process, most of them enzymes were present in the wild type but in the mutant there was a fast decay. Our biochemical results on hexokinase activities are in agreement with this observation. The strong reduction in Hxk2 protein levels during starvation was not completely reflected in drastic changes in hexokinase activity. This apparent discrepancy could be explained by the induction of *HXK1* and *GLK1*. The corresponding Hxk1 and Glk1 proteins, which would keep the capacity to phosphorylate glucose, are less abundant than Hxk2 and we did not identify them by proteomics; however, our transcriptomic results support that possibility.

On the other hand enzymes involved in two other important energetic pathways were detected: pentose phosphate and methylglyoxal pathways; in general, proteins from both pathways remained present during the starvation process. In fact, the transcriptomic profile of *GLO1* and *GLO2*, the two genes involved in detoxification of methylglyoxal, shows induction during starvation (not shown). It is tempting to speculate that the glycolysis pathway is more sensitive to low K^+^ than the alternative pathways and for that reason it is more inhibited in the mutant. It has been reported that, on the one hand, potassium plays a crucial role in the activation of the glycolytic enzyme pyruvate kinase (see Page and Di Cera (2006) for a review) and, on the other hand, the mutant shows defective potassium transport. These facts may be related to the higher sensitivity of the glycolysis in the mutant.

We have mentioned the importance of the stress induced by potassium starvation, especially in mutant cells. The fact that two ubiquitin enzymes related with DNA-repair system (Ubc2 and Mms2) were identified along the 5 h of the experiment is in agreement with this observation (Broomfield et al. 1998; Game and Chernikova 2009). Even more in the mutant, the amount of the two ubiquitin proteins was not only present, but significantly increased during potassium starvation. On the other hand, some important pathways seem to be unaffected by starvation. Two examples are the metabolism of some amino acids (methionine, lysine) and bases (pyrimidine ribonucleotides). The application of two different algorithms for sample classification, one of them based on recent algorithms based on neural networks, lead to the obtaining of complementary results increasing the discriminatory power of this analysis (Valledor and Jorrín 2011). Cluster analysis allowed a distance-based classification of the samples and spots reinforcing that idea. Five major groups of spots could be distinguished in the plot being relevant that most of the glycolytic proteins appear in groups I and II and show a completely different behavior in wild-type and mutant strain.

It is conceivable to pose the question about how *TRK* mutation affects these metabolic processes. We have no definitive answer to this question but our results indicating a defective metabolic adaptation to the lack of potassium in the mutant may be taken as a clue on the relevant role of potassium fluxes and/or levels triggering adaptation. Unpublished results of our group show that wild-type and *trk1,2* cells grown under nonlimiting KCl are able to adapt and reach a new internal K^+^ stationary state when suspended in lower K^+^ concentrations, requiring mutant cells higher external K^+^ to keep similar internal amounts of the cation. In conclusion, the DM *trk1,2* is still able to sense a decrease in external potassium but lacks the mechanism to properly adapt to this stress. A similar behavior may explain the defective metabolic adaptation during starvation.

In summary, the decrease in protein content during potassium starvation experiments lead to a global decrease of the basic cellular functions such as the cell energy production pathways, with a radical decrease of the glycolytic proteins that was more evident in the mutant. In the context of a general decrease of proteins, it is relevant that some cellular processes such as the pentose phosphate and methylglyoxal pathways were kept. These results indicate that conditions commonly used in the past to characterize adaptation to potassium (4–5 h in the absence of the cation) are too stressful for the cells and this should be considered in future studies on potassium homeostasis. In fact, the study of the proteome under less extreme potassium limitation is under way. This would allow to analyze differences between parental and mutant strains under more physiological conditions.

## Experimental Procedures

### Strains and growth conditions

*Saccharomyces cerevisiae* wild-type BY4741 (MATa*his3Δ1leu2Δ met15Δ ura3Δ*; EUROSCARF, Germany) and the derivative isogenic potassium transport DM (*trk1*,*trk2*) (BYT12, *trk1Δ::loxPtrk2Δ::loxP*) (Petrezselyova et al. 2010) strains were grown, in 250-mL Erlenmeyer flasks, with 100-mL K^+^-free translucent YNB medium: 1.63 g/L Yeast Nitrogen Base (ForMedium™ UK, CYN7505), 2% glucose, 4 g/L ammonium sulphate, enriched with 50mM potassium chloride, 20 mg/L methionine, adenine, tryptophan, and arginine; 30 mg/L tyrosine, leucine, isoleucine, and lysine; 40 mg/L uracyl, histidine; 50 mg/L phenylalanine, 100 mg/L glutamic and aspartic acid; 150 mg/L valine; 200 mg/L threonine and 400 mg/L serine. The cultures were grown at 28°C (initial OD_600nm_ = 0.05) in a shaker to allow good aeration (180 rpm), until they reach OD_600nm_ = 1.9. K^+^ starvation was induced by washing the cells twice with milli-Q water, and then resuspending in YNB medium without KCl. At indicated times, cell samples were withdrawn and used for protein extraction.

### Protein extraction

Cells were recovered by centrifugation. The cell pellet was suspended in 600 μL homogenization buffer (50 mM TRIS buffer, pH 7.6, containing 1 mM PMSF, 1 mM EDTA, 2 mM DTT, and a tablet of protease inhibitor cocktail [Roche-11697498001] per 50 mL of homogenization buffer). Cells were broken by vortexing (15–20 times, 30 sec) in the presence of glass beads (Sigma-G9268) (ratio v/v of 1). Glass beads and insoluble material were eliminated by centrifugation (10,000 *g*, 5 min). To the supernatant (about 500 μL), 1.5 mL of 10% (w/v) trichloroacetic acid (TCA)/acetone solution containing 0.07% (w/v) dithiothreitol (DTT) was added. Proteins were allowed to precipitate at –20°C for 1 h; then, the precipitate was recovered after centrifugation at 10,000 *g* for 15 min. The pellet was washed twice with 1.5 mL of cold (–20°C) acetone containing 0.07% (w/v) DTT. The protein pellet was recovered after centrifugation. The final pellet was air-dried and solubilized in 250 μL of 8 M urea, 2% (w/v) 3-[(3-cholamidopropyl)dimethylammonio]propanesulfonate (CHAPS), 20 mM DTT, 0.5% (v/v) Biolytes pH range 3–10 (Bio-Rad), and 0.0001% (w/v) bromophenol blue. Insoluble material was removed by centrifugation. The protein concentration was determined by Bradford, with ovoalbumin as a standard.

### 2-DE

Immobilized pH gradients (IPG) strips (17 cm, 5–8 pH linear gradient; Bio-Rad) were passively rehydrated for 2 h with 500 μg of protein in 300 μL of IEF solubilization buffer (7 M urea; 2 M thiourea; 4% [w/v] CHAPS; 0.5% [v/v] IPG buffer 5–8, 20 mM DTT; and 0.01% [w/v] bromophenol blue). The strips were loaded onto a Bio-Rad Protean IEF Cell System and proteins were electrofocused at 20°C with a first step of a gradual increase in the voltage (50–8000 V) and then reaching 60,000 Vh. Strips were immediately equilibrated according to Görg et al. (1987). Second dimension SDS-PAGE was performed on 12% polyacrylamide gels using Protean Dodeca Cell System (Bio-Rad). Gels were run first at 30 mA per gel for 15 min and then at 50 mA per gel until the dye front reached the bottom of the gel.

### Staining and image analysis

Gels were stained twice with CBB G-250 (Bio-Rad) for 20 h following the method described by Mathesius et al. (2001). Images were acquired with a GS-800 calibrated densitometer (Bio-Rad) and analyzed with PDQuest 8.0.1 software (Bio-Rad) using 10-fold over background as a minimum criterion for presence/absence for the guided protein spot detection method. This criterion includes almost all spots of the gels and some staining artifacts and noise. A spot-by-spot visual validation of automated analysis was done thereafter to increase the reliability of the matching. Experimental pI was determined using a 5–8 linear scale over the total length of the IPG strip. Mr values were calculated by mobility comparisons with protein standards markers (SDS molecular weight standards, Broad range, Bio-Rad) run in a separate lane in the gel.

### Statistics

Statistical analysis was performed following the recommendations proposed by Valledor and Jorrín (2011). In brief, spot volumes were preprocessed before statistical analyses. Spot volumes were first normalized as a proportion of the total spot intensities per gel (spot volume × 10^5^/Σ gel spot volumes), and then the normalized volumes were log transformed to reduce the volume-variance dependency. Spot values passed the Levene's homoscedasticity and Kolmogorov–Smirnov normality tests. Differentially abundant spots were defined after applying a two-way ANOVA considering strain and sampling time as factors. False discovery rate (FDR) *q*-values were calculated with FDRtool package. Cut-off *q*-value was set to allow less than one false positive in this study. The joint spot analysis was performed following three different multivariate approaches: PCA (centered normalized spot values, unrotated solution), heat map clustering (employing Euclidean distance and Ward's aggregation method), and a neural-network based SOM (centered values, 4 × 4, hexagonal topology).

Three biological and one technical replicates were done for each time and strain. All of the statistical analyses were performed in R Environment v 2.12 (R Development Core Team 2011) employing its core functions and the packages gplots2, Kohonen, and FDRtool.

### MS analysis and protein identification

Spots were manually excised and transferred to multiwell 96 plates. Spots were digested with bovine trypsin (sequencing grade Roche Molecular Biochemicals) using an Ettan™ digester station (GE Healthcare Life Sciences). The digestion protocol used was that of Schevchenko et al. (1996), with minor variations. Briefly, spots were washed twice with water and distained by twice 10-min incubation with 100% acetonitrile and dried in vacuum (Savant SpeedVac) for 30 min. Then the samples were reduced with 10 mM dithiotreitol in 25 mM ammonium bicarbonate for 30 min at 56°C and subsequently alkylated with 55 mM iodoacetamide in 25 mM ammonium bicarbonate for 15 min in the dark. Finally, samples were digested with 12 μL of trypsin (12.5 ng/μL) in 25 mM ammonium bicarbonate (pH 8.5) overnight at 37°C. After digestion, the supernatant was collected and 1 μL was spotted onto a MALDI target plate using the dry droplet method and 0.4 μL of a 3 mg/mL of α-cyano-4-hydroxy-transcinnamic acid matrix in 50% acetonitrile (ACN) and 0.1% trifluoroacetic acid (TFA). Samples were analyzed in a 4800 Proteomics Analyzer MALDI-TOF/TOF mass spectrometer (Applied Biosystems, Framingham, MA), in the *m/z* range 850–4000, with an accelerating voltage of 20 kV, in reflectron mode and with a delayed extraction set to 120 nsec. All MS spectra were internally calibrated with peptides from trypsin autolysis. The MS analysis by MALDI-TOF/TOF mass spectrometry produces peptide mass fingerprints and the peptides observed with a signal to noise greater than 20 can be collated and represented as a list of monoisotopic molecular weights. Proteins ambiguously identified by peptide mass fingerprints were subjected to MS–MS sequencing analysis. So, from the MS spectra suitable precursors were selected for MS–MS analysis with collision-induced dissociation (CID) on (atmospheric gas was used) 1 kV ion reflector mode and precursor mass Windows ± 5 Da. The plate model and default calibration were optimized for the MS–MS spectra processing. For protein identification, the UniProt Knowledgebase Release 14.6 (UniProtKB/Swiss-Prot Release 56.6 of 16 December 2008, Uni-ProtKB/TrEMBL Release 39.6 of 16 December 2008) was searched using MASCOT search engine v.2.1 (Matrix Science; http://www.matrixscience.com) through the Global Protein Server Explorer software v3.6 from Applied Biosystems.

The following parameters were allowed: taxonomy restrictions to *S. cerevisiae*, one missed cleavage, 50 80–100 and 50 80–100 ppm mass tolerance for peptide mass fingerprinting (PMF) and MS–MS searches, respectively, 0.3 Da for MS-MS fragments tolerance, carbamidomethylation cysteine as a fixed modification, and methionine oxidation as a variable modification. The parameters for the combined search (peptide mass fingerprint plus MS–MS spectra) were the same described above.

In all protein identified, the probability scores were greater than the score fixed by Mascot as significant with a *P*-value less than 0.05.

### Enzymatic activity determinations

Growth of cultures was as above, except that cells were washed in K^+^-free translucent medium instead of milliQ water. Whole-cell lysates (25 mL of culture) were prepared by resuspending the cells in 500 μL of homogenization buffer (20 mM imidazole, pH 7.0). One volume of 0.5-mm zirconia/silica beads (Biospec Products, Inc.) was added and cells were broken at 4°C by vigorous shaking in a Fastprep-24 cell breaker (MP Biomedicals) for five times (30 sec each, setting 5), with intervals of 1 min on ice. After sedimentation at 1000 × *g* for 15 min at 4°C, the cleared lysate was recovered and the protein concentration was determined by the Bradford assay.

The hexokinase activity was determined as described in Gancedo et al. (1977) adding either 10 mM glucose or 25 mM fructose as substrate. The alcohol dehydrogenase activity was determined essentially as described in Ganzhorn et al. (1987) using 0.5 mM NAD^+^ and 100 mM ethanol. All the enzymatic activity measurements were performed on 96-well microplates, with a final volume of 300 μL. The reactions were monitored by following the changes in absorbance at 340 nm using a microplate-based UV spectrophotometer (Multiskan Ascent, ThermoLabsystems).

## Supporting information

Disclaimer: Supplementary materials have been peer-reviewed but not copyedited.

**Table S1**. Quantitation, univariate statistics, and identification table.

**Table S2**. PCA loading matrix.

**Table S3**. PCA score matrix.

**Table S4**. Detailed list of identified proteins.
